# Cost and Complications in Rheumatoid Arthritis Patients Undergoing Primary Hip Arthroplasty: A National Inpatient Sample-Based Study

**DOI:** 10.7759/cureus.30483

**Published:** 2022-10-19

**Authors:** Vishaal Sakthivelnathan, Akshay Goel, Philip A Serbin, Prabhudev Prasad Purudappa, Sushrruti Varatharaj, Varatharaj Mounasamy, Senthil Sambandam

**Affiliations:** 1 School of Medicine, University of Texas Medical Branch at Galveston, Galveston, USA; 2 Orthopedic Surgery, Marshall Orthopaedics, Huntington, USA; 3 Orthopedics, University of Texas Southwestern Medical Center, Dallas, USA; 4 Orthopedics, VA Boston Medical Center, Boston, USA; 5 Orthopedics, Burrell College of Osteopathic Medicine, Las Cruces, USA

**Keywords:** postoperative complication, complication, length of hospital stay (los), nationwide inpatient sample (nis), rheumatoid arthriitis, total hip arthroplasty: tha

## Abstract

Introduction: Rheumatoid arthritis (RA) is an autoimmune disease that affects multiple synovial joints in the body, including the hip. Hip involvement in RA patients is fairly common, but the current literature is lacking large-scale studies on the surgical outcomes of RA patients undergoing total hip arthroplasty (THA). The aim of the study is to examine the outcomes and hospital costs associated with THA in patients with RA and compare them to patients without RA using the National Inpatient Sample (NIS) database.

Methods: We analyzed the NIS database to identify patients undergoing THA between 2016 and 2019 using International Classification of Diseases, Tenth Revision, Clinical Modification (ICD-10-CM) diagnosis codes. Multiple variables including demographics, medical comorbidities, total hospital costs, length of stay, and perioperative complication rates were then compared between patients with and without RA. Further, the two groups were matched for demographic differences, if any, using a 1:1 propensity match algorithm.

Results: Patients with RA undergoing THA were significantly younger and predominantly female when compared to patients without RA. There was also a lower incidence of obesity and the percentage of elective THA procedures were smaller in the RA group. The RA group had a longer length of stay and increased incidences of blood loss anemia, blood transfusion, and periprosthetic fractures. These differences persisted despite matching the two groups for demographic differences, elective procedures, diabetes, obesity, and tobacco usage.

Conclusion: THA in RA is associated with an increased incidence of blood loss anemia, blood transfusion, and periprosthetic fractures, as well as a longer length of stay in THA patients.

## Introduction

Rheumatoid arthritis (RA) is a chronic autoimmune disorder characterized by inflammation of the synovium [1]. Conservative estimates suggest that RA affects around 0.5-1% of the US population, with an incidence of 40 per 100,000 [2,3]. Classically, RA affects the joints of the hand, wrists, and knees. However, the current literature suggests that the prevalence of hip involvement in RA patients ranges from 18% to 33% [4-7].

Although medical management is the preferred first-line treatment, total joint replacement can be used in cases with persistent arthritic pain and stiffness. A study by Zhou et al. found the eight-year incidence rate of total hip arthroplasties (THAs) after the introduction of disease-modifying antirheumatic drugs (DMARDs) to be 7.9 per 1000 [8]. It is believed that RA patients are at higher rates of complications than osteoarthritis (OS) due to the underlying systemic inflammatory process, DMARD therapy, degree of deformity, or a combination of these factors [9]. Perioperative complications have not been well documented in RA patients and show conflicting evidence [10]. Additionally, most of the current studies focus on total knee arthroplasty or combine the outcomes of total hip and total knee arthroplasty patients into a single study cohort. The purpose of this study is to utilize the National Inpatient Sample (NIS) database to evaluate complication rates and clinical outcomes in RA patients undergoing THA as compared to RA-negative patients.

## Materials and methods

The NIS database, a component of the Healthcare Cost and Utilization Project (HCUP), was used to stratify THA patients between the years 2016 and 2019. It is the largest database that contains all-payer inpatient care data in the US, encompassing 20% of the hospitals in the US. An independent contractor verifies the data through a quality assessment evaluation that compares data points to standardized normative values.

The NIS includes information regarding demographics, length of stay, total charges, payment source, discharge status, comorbidities, and many perioperative outcome variables. Data from 2016 to 2019 were selected as 2016 was the year the NIS switched to the International Classification of Diseases, Tenth Revision, Clinical Modification/Procedure Coding System (ICD-10-CM/PCS).

Data acquisition

This study was exempt from IRB approval since the data were publicly available, deidentified data. THA patients were identified using the ICD-10 procedure codes OSR8 and OSR9. Patients were classified into two groups: those with RA (M06.9) and those without RA.

Demographic variables analyzed include 1) age, 2) sex, 3) ethnicity, 4) obesity, 5) elective status, 6) diabetes, 7) tobacco use, and 8) obesity (Table 1). Perioperative outcome variables included 1) mortality, 2) acute renal failure, 3) myocardial infarction, 4) blood loss anemia, 5) pneumonia, 6) pulmonary embolism, 7) deep vein thrombosis, 8) periprosthetic fracture, 9) periprosthetic dislocation, 10) periprosthetic mechanical complications, 11) periprosthetic infection, 12) superficial surgical site infection, 13) deep surgical site infection, 14) wound dehiscence, and 15) blood transfusion rates.

**Table 1:** Ethnicity of patients RA, rheumatoid arthritis.

	RA group	Control group
Caucasian	86.1%	67%
African American	7.7%	12%
Hispanic	3.5%	13%
Asian	0.9%	4%
Native American	0.3%	1%
Other	1.6%	3%

Statistical analysis

SPSS version 27.0 was used for conducting statistical analysis (IBM, Armonk, NY, USA). An unmatched analysis and matched analysis using a 1:1 propensity matching algorithm using the preoperative were performed. Numerical variables were analyzed using t-tests and binomial variables were analyzed using chi-squared analyses. Fischer's exact tests were used when the incidence values were less than 5. Statistical significance was defined as p <0.05. Odds ratios and the corresponding 95% confidence intervals for the surgical outcomes and complications were measured as a ratio of the incidence in the RA group to the incidence in the RA negative control group. 

## Results

Demographic data

A total of 591 THA patients had RA and 308,394 patients did not have RA. The average age of the RA group was 60.63 years, as compared to 65.95 (p < 0.001) in the control group (Table 2). The RA group had a higher proportion of females (78.4% vs 55.8%, p < 0.001), a smaller proportion of the THAs as elective procedure (95.4% vs 97.1%, p = 0.014), and a smaller proportion of obesity (17.8% vs 22.9%, p = 0.003). All the other demographic variables were not statistically significant. 

**Table 2:** Patient demographics RA, rheumatoid arthritis.

	RA group	Control group	Odds ratio (RA group/control group)	95% confidence interval	Significance
Preoperative variables					
Mean age (standard deviation) in years	60.63 (14.211)	65.95 (10.647)	-----------------	------------------	p < 0.001
Sex (percentage female)	78.5%	55.8%	2.9	[2.38, 3.53]	p < 0.001
Elective versus non-elective admission (percentage elective)	95.4%	97.1%	0.61	[0.42, 0.90]	p = 0.014
Diabetes without complication (percentage diabetic)	9.31%	11.5%	0.89	[0.68, 1.18]	p = 0.495
Tobacco use disorder (percentage users)	14.0%	18.2%	0.74	[0.58, 0.93]	p = 0.10
Obesity (percentage obese)	17.8%	22.9%	0.73	[0.59, 0.90]	p = 0.003

Unmatched postoperative outcomes analysis

Patients with RA undergoing THA had a higher incidence of blood loss anemia compared to the non-RA group, 26.2% vs 17.2%, p < 0.01 (Table 3). Additionally, the incidence of blood transfusions was higher in the RA group (8.5% vs 2.2%, p < 0.001). Rates of periprosthetic fractures were also higher in the RA group as compared to the non-RA group (1.9% vs 0.5%, p < 0.01). All other postoperative complication variables were not statistically significant.

**Table 3:** Unmatched analysis RA, rheumatoid arthritis.

	RA group	Control group	Odds ratio (RA group/control group)	95% confidence interval	Significance
Postoperative variables (Incidence percentage)					
Mortality	0.17%	0%	5.806	[0.808, 41.737]	p = 0.160
Acute renal failure	1.5%	1.6%	0.936	[0.484, 1.808]	p = 1.000
Myocardial infarction	0%	0%	1	[1, 1]	p = 1.000
Blood loss anemia	26.2%	17.2%	1.710	[1.424, 2.055]	p < 0.01
Pneumonia	0.17%	0.12%	1.389	[0.195, 9.903]	p = 0.514
Pulmonary embolism	0%	0%	1	[1, 1]	p = 1.000
Deep vein thrombosis	0.17%	0%	2.214	[0.310, 15.809]	p = 0.365
Periprosthetic fracture	1.86%	0.53%	3.529	[1.940, 6.421]	p < 0.01
Periprosthetic dislocation	0.17%	0.17%	0.972	[0.136, 6.925]	p = 1.000
Periprosthetic mechanical complication	0%	0%	1	[1, 1]	p = 1.000
Periprosthetic infection	0.17%	0%	24.898	[3.344, 185.400]	p = 0.041
Superficial surgical site infection	0%	0%	1	[1 ,1]	p = 1.000
Deep surgical site infection	0%	0%	1	[1 ,1]	p = 1.000
Wound dehiscence	0.17%	0%	23.767	[3.198, 176.606]	p = 0.043
Blood transfusion	8.46%	2.16%	4.177	[3.123, 5.586]	p < 0.001

The average length of stay for THA patients with RA, 2.48 days with a standard deviation of 1.97 days, was greater than that for THA patients without RA, 1.97 days with a standard deviation of 1.63 days (p < 0.001). The average total incurred charges in the RA group, $70,086.32 with a standard deviation of $44,356.26, is higher than that for THA patients without RA, $62,056.19 with a standard deviation of 37,958.50 (p < 0.001). 

Matched postoperative outcomes analysis

All the postoperative outcome complication variables in the unmatched analysis were significant in the matched analysis (Table 4). The incidence of blood loss anemia in the RA group was 26.2% as compared to 20.8% in the control group (p = 0.032). The rates of blood transfusions in the RA group were 8.46% as compared to 4.72% in the control group (p = 0.013). The incidence of periprosthetic fractures in the RA group was 1.9% as compared to 0.17% in the control group (p = 0.006). All other postoperative complication variables were not statistically significant.

**Table 4:** Matched analysis RA, rheumatoid arthritis.

	RA group	Control group	Odds ratio (RA group/control group)	95% confidence interval	Significance
Postoperative variables (Incidence percentage)					
Mortality	0	0	1	[1, 1]	p = 1.000
Acute renal failure	1.52%	1.40%	1.090	[0.418, 2.846]	p = 0.100
Myocardial infarction	0%	0.17%	1	[1, 1]	p = 1.000
Blood loss anemia	26.2%	20.8%	1.353	[1.030, 1.778]	p = 0.032
Pneumonia	0.17%	0%	1	[1, 1]	p = 1.000
Pulmonary embolism	0%	0.17%	1	[1, 1]	p = 1.000
Deep vein thrombosis	0.17%	0.17%	0.968	[0.060, 15.510]	p = 1.000
Periprosthetic fracture	1.9%	0.17%	10.829	[1.394, 84.152]	p = 0.006
Periprosthetic dislocation	0.17%	0.34%	0.483	[0.044, 5.342]	p = 0.619
Periprosthetic mechanical complication	0%	0%	1	[1 ,1]	p = 1.000
Periprosthetic infection	0.17%	0%	1	[1, 1]	p = 1.000
Superficial surgical site infection	0%	0%	1	[1 ,1]	p = 1.000
Deep surgical site infection	0%	0%	1	[1 ,1]	p = 1.000
Wound dehiscence	0.17%	0%	1	[1, 1]	p = 1.000
Blood transfusion	8.46%	4.72%	1.866	[1.151, 3.024]	p = 0.013

The average length of stay for THA patients with RA, 2.58 days with a standard deviation of 1.97 days, was greater than that for THA patients without RA, 2.13 days with a standard deviation of 1.83 days (p = 0.002). The average total incurred charges in the RA group, $70,086.32 with a standard deviation of $44,356.26, was not statistically different compared to that of THA patients without RA, $72,553.33 with a standard deviation of 48,001.06. 

## Discussion

The unmatched analysis showed that RA patients who underwent THA had a significantly higher incidence of blood loss anemia, blood transfusions, and periprosthetic fractures as compared to THA patients without RA. Since these were also significant in the propensity-matched analysis, this suggests that preoperative demographic variables were not the underlying factors for the differences seen. The length of stay was greater for RA patients.

The current literature regarding the length of stay and hospitalization costs in relation to RA status in THA patients is limited and conflicting. A retrospective study by Burn et al. found that the total length of stay was increased in total hip and knee arthroplasty patients who had RA [11]. A retrospective single-center study by Kremers et al. found that hospitalization costs were similar in patients with degenerative and inflammatory arthritis of the knee [12]. However, a study by Stundner et al. found that the length of stay and cost of hospitalization were higher in total knee arthroplasty patients with RA [13]. In a retrospective single-center study by Morse et al., the authors explain that female sex, use of opiates preoperatively, and need for blood transfusions were higher in RA patients, and contribute to the difference in length of stay [14]. Our study also found that RA patients required more blood transfusions, but both the unmatched analysis and the matching analysis that used sex as a variable found a significant difference in length of stay.

RA has also been associated with an increased risk for blood loss anemia and blood transfusions for patients undergoing total joint arthroplasty. The Stundner et al. study found that blood transfusions occurred in 23.3% of RA patients undergoing total knee arthroplasty as compared to 16.6% in patients without RA [13]. Salah et al. also found RA to be a risk factor for increased blood transfusions in patients undergoing THA [15]. One reason for the increased rate of blood transfusions could be the incidence of preoperative anemia in RA patients. Data from a study by Wilson et al. showed that mild anemia is prevalent in 33-60% of patients with RA [16]. A study by Salt et al. found that the geographical location of the procedure, history of anemia, and female sex were risk factors for blood loss in RA patients undergoing total joint arthroplasty. Additionally, they found that RA patients undergoing THA were at higher risk for blood transfusion than RA patients undergoing TKA, suggesting that transfusion outcomes from TKA studies with RA patients reporting transfusions can serve as a conservative reference point for RA patients undergoing THA [17]. Morse et al. reported that tranexamic acid, an antifibrinolytic drug that has been increasingly used in total joint patients with OS to reduce postoperative infusion, does not reduce the risk of transfusion in total joint patients with RA [18]. This finding in combination with the increased risk for blood transfusions found in our study suggests a challenging course of management and a need for further studies evaluating blood loss anemia and management in RA patients undergoing THA.

To our knowledge, our study is one of the few, if any, large epidemiological studies to affirm the increased risk of periprosthetic fractures in RA patients undergoing THA. Factors such as poor bone quality, multiple joint involvement, and the extent of inflammatory disease in RA have been linked to increased risk of intraoperative and periprosthetic fractures [19,20]. The current literature contains case reports and small sample studies suggesting an increased incidence of supracondylar periprosthetic fracture after total knee replacement in RA patients, but this data for THA is sparse [20-22]. Future studies are needed to further investigate the underlying pathological mechanisms leading to an increased risk of periprosthetic fracture in RA patients undergoing THA.

This study is not without limitations. The NIS database includes information only on inpatient outcomes and does not include any data beyond the patients' hospital stay. Any conclusions drawn based on this are limited in scope since they do not analyze longer-term complications. Further, this data also does not include functional data or patient-reported outcomes data. Also, the NIS database only represents 20% of the community hospitals in the US. Despite this, the high volume of data strengthens its generalizability to the US population. NIS database also relies on accurate coding and is as such limited by the risk of incomplete data collection. However, our large-scale database study provides information that can aid in healthcare policy development and decision-making for patients, surgeons, and lawmakers regarding THA patients with RA, a population that currently has a dearth of studies in the current literature.

## Conclusions

Our study found that the presence of RA increases the risk of blood loss anemia and blood transfusion, the risk for periprosthetic fractures, and the total length of stay in THA patients. More large-scale studies are needed to further identify these findings as well as examine the underlying pathophysiological mechanisms behind them so that surgeons are better prepared for treating this patient population.

## Appendices

**Table 5:** ICD 10 codes used

Total hip arthroplasty procedure code	Rheumatoid arthritis	Obese codes	Comorbidities codes	Medical complication codes	Surgical complication codes
Replacement of left hip joint OSR8 Replacement of right hip joint OSR9	B20 B21 B22 B23 B24 B25	E660 E6601 E6609 E661 E662 E668 E669 Z6830 Z6831 Z6832 Z6833 Z6834 Z6835 Z6836 Z6837 Z6838 Z6839	Diabetes without complications E119 Diabetes with complications E1169 Tobacco-related disorder Z87891	Acute renal Failure N170, N171, N172, N178, N179; Myocardial Infarction I2101, I2102, I2111, I2113, I12114, I12119, I2121, I12129, I21A1; Blood loss anemia D62; Pneumonia J189, J159, J22; Blood transfusion 30233N1; Pulmonary embolism I2602, I2609, I2692, I2699; Deep vein thrombosis I82401, I82402, I82403, I82409, I82411, I82412, I82413, I82419, I82421, I82422, I82423, I82429, I82431, I82432, I82433, I82439, I82441, I82442, I82443, I82449, I82491, I82492, I82493, I82499, I824Y1, I824Y2, I824Y3, I824Y9, I824Z1, I824Z2, I824Z3, I824Z4	Periprosthetic fracture T84010A, T84011A, T84012A, T84013A, T84018A, T84019A, M9665, M96661, M96662, M96669, M96671, M96672, M96679, M9669, M9701XA, M9702XA, M9711XA, M9712XA; Periprosthetic dislocation T84020A, T84021A, T84022A, T84023A, T84028A, T84029A; Periprosthetic mechanical complications, Periprosthetic fracture T84090A, T84091A, T84092A, T84093A, T84098A, T84099A; Periprosthetic Infection T8450XA, T8451XA, T8452XA, T8453XA, T8454XA, T8459XA; Superficial surgical site infection T8141XA; Deep surgical site infection T8142XA; Wound dehiscence T8130XA, T8131XA, T8132XA
